# Advanced progress in the genetic modification of the oncolytic HSV-1 virus

**DOI:** 10.3389/fonc.2024.1525940

**Published:** 2025-01-21

**Authors:** Mi Zhou, Zhenyu Shen

**Affiliations:** ^1^ Department of Stomatology, Union Hospital, Tongji Medical College, Huazhong University of Science and Technology, Wuhan, China; ^2^ Hubei Province Key Laboratory of Oral and Maxillofacial Development and Regeneration, Wuhan, China; ^3^ School of Stomatology, Tongji Medical College, Huazhong University of Science and Technology, Wuhan, China

**Keywords:** oncolytic virotherapy, herpes virus 1, genetical engineering, solid tumor, genetic modification, cancer treatment

## Abstract

The use of replication-competent viruses for selective tumor oncolysis while sparing normal cells marks a significant advancement in cancer treatment. HSV-1 presents several advantages that position it as a leading candidate for oncolytic virotherapies. Its large genome can accommodate insertions over 30 kb or deletions of multiple virulence genes without compromising lytic replication in tumor cells. Additionally, anti-herpes drugs can inhibit its replication during accidental infections. Importantly, HSV-1 does not integrate into the host genome and cause mutations. The HSV-1 genome can be modified through genetic engineering in two main ways: first, by reducing infectivity and toxicity to normal cells via limited replication and assembly, altered protein-virus receptor binding, and minimized immune evasion; second, by enhancing anticancer activity through disruption of tumor cell metabolism, induction of autophagy, improved immune recognition, and modification of the tumor microenvironment. In this mini-review, we systematically examine genetic modification strategies for oncolytic HSV-1 while highlighting advancements from these modifications. Certain genetic alterations have shown efficacy in improving clinical outcomes for HSV-1-based therapies. These modifications include silencing specific genes and inserting exogenous genes into the HSV-1 genome. The insertion of exogenous genes has increasingly been used to develop new oncolytic HSV-1 variants. Finally, we discuss limitations associated with oncolytic virotherapy at the conclusion of this review. As more clinical trials explore newly engineered therapies, they are likely to yield breakthroughs and promote broader adoption for cancer treatment.

## 1 Introduction

Recent advancements in cancer treatment include the utilization of replication-competent viruses for selective oncolysis of tumors, known as oncolytic viruses (OVs), while sparing normal cells. This therapeutic approach can be employed either as a standalone treatment or in combination with other therapies to inhibit tumor progression (1). OVs encompass both wild-type and genetically modified variants. Genetic engineering strategies aimed at modifying these viruses involve deleting specific genes to limit toxicity to healthy cells (2, 3), inserting genes to activate the immune system, stimulate immune responses, or inhibit angiogenesis (4–6), and combinations of these strategies.

Herpes simplex virus type 1 (HSV-1) possesses a genome consisting of 152 kb of double-stranded linear DNA that encodes approximately 85 protein-coding genes, with 47 being dispensable in cell culture (7). HSV-1 has several advantages that position it as a leading candidate for oncolytic virotherapy (OVT). Notably, its genome contains two unique segments: one is the unique long (UL) segment and the other is the unique short (US) segment; each is flanked by inverted repeat (IR) elements. This genomic architecture allows for the insertion of fragments exceeding 30 kb or deletion of multiple virulence genes without compromising its lytic replication cycle within tumor cells (8, 9). Additionally, anti-herpetic drugs can inhibit HSV-1 replication in cases of accidental infection (10). Importantly, HSV-1 does not integrate into the host genome nor induce insertional mutations (7). Due to the above characteristics of HSV-1 virus, it has three advantages compared to other oncolytic viruses. First, it has a larger genome that can insert and accommodate multiple foreign genes. Additionally, the use of acyclovir can easily control HSV-1 infections in non-tumor cells. Finally, theoretically, HSV-1 has a lower likelihood of causing insertional mutagenesis in infected cells.

**Figure 1:**
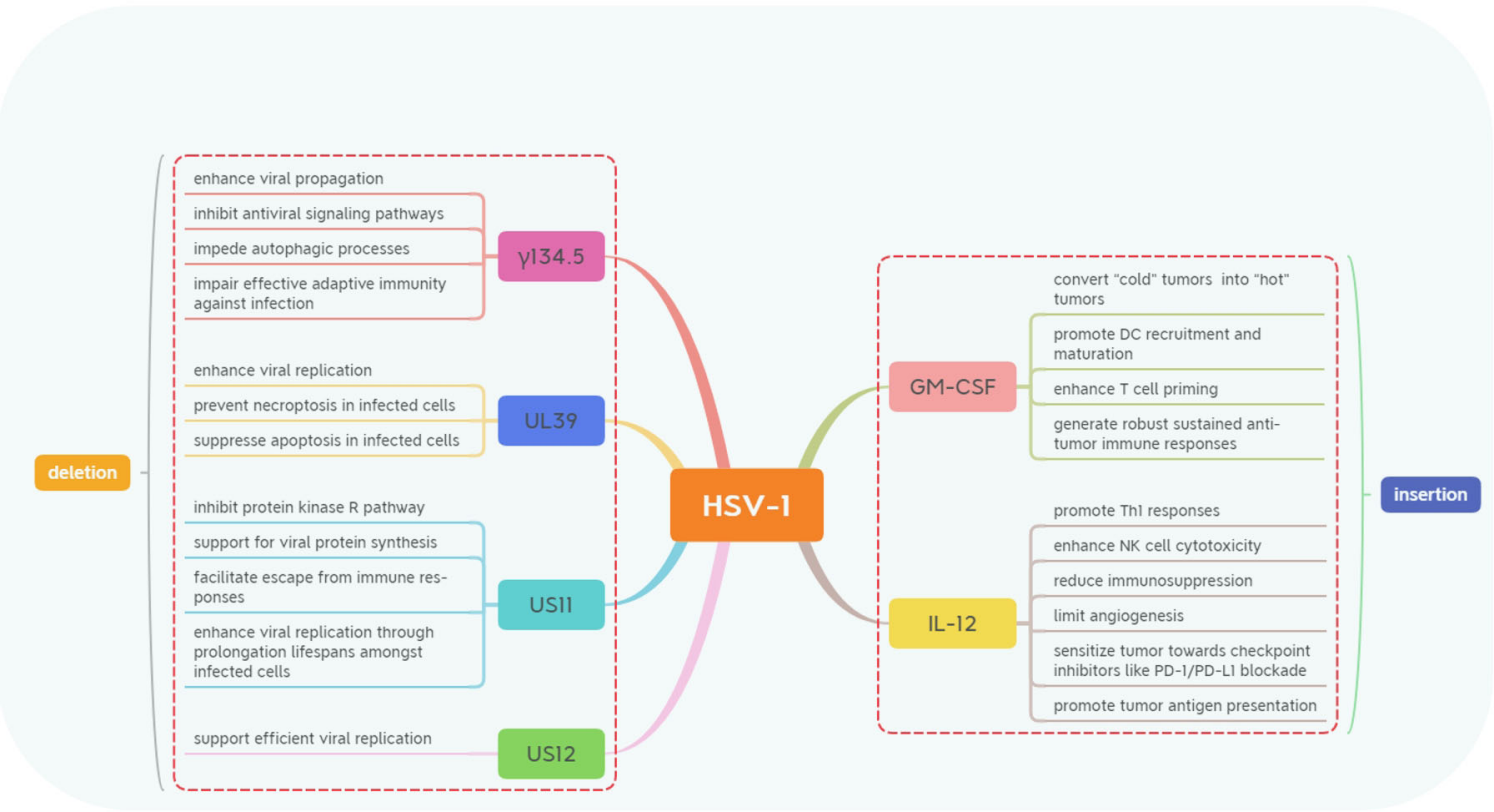
The genetic modifications in Herpes Simplex Virus-1 (HSV-1) involve deletions and insertions. These majority modifications include the deletion of genes such as γ134.5, US11, US12, and UL39 and the expression of transgenes like GM-CSF and IL-12. These strategic genetic engineering techniques are designed to enhance the oncolytic properties of HSV-1 while modulating immune responses to improve anti-tumor efficacy through various mechanisms.

Currently, there are two primary directions for genetic modification of oncolytic HSV-1 (**Figure 1**). The first direction focuses on reducing HSV-1’s infectivity and toxicity towards normal cells by limiting viral replication and assembly (3, 11–13), modifying proteins that bind viral receptors (14), and decreasing mechanisms involved in viral immune evasion (15, 16). The second direction aims to enhance HSV-1’s anticancer efficacy through interference with tumor cell metabolism (17), induction of autophagy (18, 19), improvement in immune recognition processes (20–23), and alteration of the tumor microenvironment itself (6, 24, 25). Several key gene modifications related to silencing specific genes or introducing exogenous genes into the HSV-1 genome will be discussed separately.

## 2 Silencing genes

### 2.1 Gene γ134.5

To enhance the selectivity of HSV-1 for infecting epithelial-derived malignancies while minimizing the risk of infection in healthy somatic cells and preventing uncontrolled spread of HSV-1 to normal somatic cells, numerous research groups knockout the γ134.5 gene. The diploid gene γ134.5 located within the inverted terminal repeats flanking the long unique sequence of HSV-1 DNA is classified as a gamma-late or “leaky late” gene. It encodes ICP34.5, a neurovirulent protein. ICP34.5 consists of 263 amino acids organized into three main domains: the N-terminal domain, the linker region, and the C-terminal domain. Those domains are responsible for binding host proteins essential for both viral replication and immune evasion (26–29).

The principal function attributed to ICP34.5 involves enhancing viral propagation across peripheral tissues alongside central nervous systems, contributing significantly toward HSV, and inducing neurovirulence via various mechanisms including protein phosphatase I (PPI) dephosphorylating eIF2α, thus preventing shutoff from host protein synthesis while enabling continuous production (11, 30, 31). Furthermore, ICP34.5 converts proliferating cell nuclear antigen (PCNA) from repair mode back toward a replicative state, crucially initiating HSV replication (32).

Moreover, ICP34.5 inhibits antiviral signaling pathways ensuring persistent infections. ICP34.5 disrupts retinoic-acid-inducible gene I (RIG-I) signaling preventing interaction between RIG-I and mitochondrial antiviral signaling protein (MAVS), a pivotal adaptor inhibiting downstream activation IRF3 and subsequent IFN production (33). Stimulator interferon genes (STING) is another important player during antiviral responses where N-terminal domain binds/inactivates STING, thereby diminishing IRF3 activation/IFN secretion (34).

Additionally, ICP34.5 impedes autophagic processes through Beclin binding interactions specifically targeting Beclin-1 (Atg6) (35). Such engagement hinders this vital cellular defense mechanism allowing enhanced pathogenesis while blocking class II antigen presentation, further augmenting HSV virulence (36, 37).

Lastly, ICP34.5 also disrupts NF-kB activation suppressing dendritic maturation and ultimately impairing effective adaptive immunity against infection. The N-terminal domain of ICP34.5 interacts with IKKα/β, components of the IκB kinase complex, while its C-terminal domain recruits PP1α. This interaction leads to the dephosphorylation of IκB kinase, preventing the activation of NF-κB, a transcription factor that regulates genes involved in immune responses, inflammation, and cell survival (38, 39).

Through these combined mechanisms, ICP34.5 serves as a critical factor in HSV-1 pathogenesis by supporting viral replication, evading multiple immune pathways, and altering host cellular functions. However, after silent gene γ134.5, the oncolytic efficacy of HSV-1 in malignant tumors of neurological origin (e.g., glioblastoma and neurofibroma) has decreased. In clinical treatment, it is necessary to choose the oncolytic HSV-1 with silent gene γ134.5 according to the tissue source of the tumor.

### 2.2 Gene US11

To reduce immune evasion and subsequent uncontrolled viral infection after the injection of oncolytic HSV-1 into patients, gene US11 was selectively silenced. It can not only reduce the immune evasion of oncolytic HSV virus immunocompromised patients but also decrease the replication and spread of the virus in healthy cells. The US11 protein is a small basic phosphoprotein with a molecular weight of approximately 18 kDa. Its coding region extends from the ATG codon at residue 12,641 to the TAG stop codon at residue 12,158, resulting in a protein mass of 17,756 Da. The carboxy-terminal half contains several arginine-X-proline (R-X-P) repeats that confer RNA-binding capability (40). These repeats also harbor nucleolar import and nuclear export signals that facilitate localization within both nucleus and cytoplasm as required. Encoded by the late γ2 gene, US11 is expressed during later stages of HSV infection and performs several crucial functions enhancing HSV-1 survival within host cells (41).

Inhibition of protein kinase R (PKR) pathway alongside support for viral protein synthesis are primary functions attributed to US11. The PKR pathway becomes activated upon binding double-stranded RNA (dsRNA), leading to phosphorylation of eIF2α, an event typically halting protein synthesis as part of an antiviral response. US11 exhibits high affinity for dsRNA, allowing it to sequester this molecule away from PKR (42, 43). By obstructing PKR activation through this mechanism, US11 prevents eIF2α phosphorylation, thus sustaining viral protein synthesis. Furthermore, when expressed early during infection, US11 can partially compensate for ICP34.5’s function inhibiting eIF2α phosphorylation. This redundancy enables HSV-1 to maintain ongoing translation even if ICP34.5 is absent. However, both proteins are generally necessary for full resistance against type I interferon (IFN) responses (43).

Additionally, US11 modulates various host antiviral pathways facilitating escape from immune responses by HSV-1. During late phases when levels of dsRNA peak, US11 binds/sequesters dsRNA effectively preventing MDA5/RIG-I activations, which subsequently suppress IRF3 activity along with interferons production (44, 45). Such inhibition impedes induction of ISGs establishment, hence compromising antiviral states among infected cells. Another important mechanism involves oligoadenylate synthetase (OAS) pathway activated via dsRNA binding where US11 inhibits OAS activity, blocking RNase L, thereby aiding virus evade degradation while preserving infectivity (46).

Moreover, US11 plays pivotal roles regulating cell survival pathways ultimately promoting enhanced replication through prolongation of lifespans among infected hosts. Within nuclei, US11 interacts with homeodomain-interacting protein kinase HIPK2 responding stress signals including those arising from ER-regulating cycle progression/pro-apoptotic signaling (47). By antagonizing growth-arrest-induced HIPK2, HSV-1-infected cells evade apoptosis, continuing to facilitate virion propagation (48).

Through multifaceted functionalities, US11 facilitates HSV-1 replication by preventing translational shutoff, inhibiting immunological signaling and obstructing pro-apoptotic response. By suppressing activations across PKR, OAS, MDA5, and RIG-I enable HSV-1 to evade defenses and sustain syntheses, thus augmenting survivability and pathogenicity. After the silencing of US11, the therapeutic effect of a single injection of oncolytic HSV-1 may be transient. Throughout the course of treating malignant tumors, multiple injections of oncolytic HSV-1 are required, and continuous monitoring of tumor growth is necessary to evaluate whether to administer oncolytic HSV-1 again.

### 2.3 Gene US12

One of the immune evasion mechanisms of HSV-1 is to inhibit antigen presentation by binding to TAP, thereby preventing cytotoxic T cells from recognizing infected cells. The protein encoded by the US12 gene is key to binding with TAP. The US12 gene (ICP47) spans residues 12,972 to 12,708 and encodes the immediate-early protein ICP47, which consists of 88 amino acids. Similar to US1 at the opposite end of the Us region, both the promoter region of ICP47 and a significant portion of its 5′-non-coding mRNA are situated within the terminal repeat (TR) sequences of HSV-1 (26). ICP47 plays a pivotal role in HSV-1’s immune evasion strategy through various mechanisms and polymorphic functions during different stages of infection. It exhibits high-affinity binding to the transporter associated with antigen presentation (TAP). By occupying TAP’s substrate-binding site, ICP47 inhibits viral peptide loading onto MHC class I molecules for presentation on cell surfaces to CD8+ T cells, effectively blocking cytotoxic T lymphocyte (CTL) recognition of infected cells and enabling HSV-1 to evade immune detection (14, 49).

The function of ICP47 is polymorphic as infection progresses. During early infection stages, it may impede RNA splicing, thus limiting host and viral gene expression in a tightly regulated manner. In later stages, however, ICP47 appears to facilitate viral mRNA export from the nucleus into the cytoplasm, thereby supporting efficient viral replication (50).

Deletion of US12 has been shown to enhance HSV-1’s oncolytic potential and tumor-cell-killing ability alongside a stronger immune response. This deletion places US11 under immediate-early promoter control while enhancing replication efficiency in tumor cells for HSV-1 strains lacking ICP34.5, suggesting promising applications for oncolytic virotherapy (51, 52).

In summary, when ICP47 is deleted from its genome context, it can serve as an effective tool to reduce immune evasion in immunodeficient environments and tumor cells.

### 2.4 Gene UL39

The UL39 gene is situated within the Unique Long Region of the HSV-1 genome and plays a critical role in viral replication and in modulating physiological processes within host cells (53). To reduce the spread of oncolytic HSV-1 proliferation within tumors, gene UL39 is selected as the candidate gene to be silenced. Unlike certain other HSV-1 genes, UL39 does not generate repetitive sequences with adjacent regions of the viral genome, rendering it structurally distinct. This gene is expressed early during the HSV-1 replication cycle, prior to the entry of the viral genome into the host cell nucleus (54, 55). Its initial translation depends on transcription and translation mechanisms within host cells, enabling HSV-1 to swiftly produce essential proteins for sustained infection (55).

ICP6, which is encoded by the UL39 gene and serves as the large subunit of ribonucleotide reductase, is vital for converting ribonucleotides into deoxyribonucleotides necessary for DNA synthesis in viruses (56). Additionally, ICP6 can phosphorylate eIF2α, a key initiation factor, thereby suppressing host protein synthesis and favoring production of viral proteins over cellular functions. This mechanism facilitates enhanced viral replication within infected hosts (57).

Another significant function attributed to ICP6 involves its modulation of programmed cell death (PCD) processes in infected cells through its receptor-interacting protein-homotypic interaction motif (RHIM) (54). The RHIM domain prevents necroptosis by obstructing RIPK1-RIPK3 complex formation (receptor-interacting protein kinases 1 and 3) in human cells (58). Furthermore, it promotes aggregation of RIPK1 that subsequently undergoes degradation via aggrephagy, further diminishing necroptotic activity (11). It also inhibits RIPK1/RIPK3-dependent necroptosis in human cells. Beyond preventing necroptosis, ICP6 additionally suppresses apoptosis by directly binding to and inhibiting caspase-8. This dual inhibition strategy allows HSV-1 to circumvent major apoptotic pathways while promoting both survival and proliferation within host environments (53).

Inactivation of ICP6 through fusion with LacZ results in restricted virus propagation primarily among dividing cells, particularly tumor cells capable of supplying deoxyribonucleotides via endogenous pathways (59). This tumor-specific characteristic exhibited by mutated forms of ICP6 positions HSV-1 variants makes them promising candidates for oncolytic therapies. Although silencing the gene UL39 can limit the proliferation of oncolytic HSV-1 after injection, enhancing the safety of this oncolytic virus in clinical applications, the tumor-killing effect of this oncolytic virus is also restricted, requiring a larger dosage and multiple injections to achieve the desired effect.

## 3 Inserting exogenous genes

### 3.1 GM-CSF

Induction of immune cells to kill tumor cells is one of the key mechanisms of oncolytic virus anticancer. In addition to the immune activation of the viral particles themselves, the cytokine genes carried by oncolytic viruses can be synthesized and released in tumor cells, and this process also significantly improves the killing efficacy of immune cells to tumor cells. Granulocyte-macrophage colony-stimulating factor (GM-CSF) is a multifunctional cytokine that plays critical roles in immune modulation, serving as a bridge between hematopoiesis and immune activation (60, 61). Initially identified as a growth factor that stimulates the differentiation of bone marrow progenitor cells into granulocytes and macrophages, GM-CSF also activates various signaling pathways, including JAK/STAT, MAPK, and PI3K, through JAK2 activation, thereby influencing immune functions (62–65).

GM-CSF enhances the survival, proliferation, and differentiation of myeloid lineage cells such as neutrophils, macrophages, and dendritic cells (DCs) (66). By promoting DC maturation, GM-CSF improves antigen presentation capabilities and T-cell activation (60). To enhance the phagocytic abilities of macrophages and their anti-tumor activities, GM-CSF drives the polarization of these cells from an M2 (anti-inflammatory) phenotype to an M1 (pro-inflammatory) phenotype (67). Furthermore, GM-CSF strengthens immune recognition of cancer cell neoantigens by fostering antigen, presenting cell generation, and elevating major histocompatibility complex (MHC) expression, thereby reinforcing the overall immune response against tumors (23, 68).

Oncolytic viruses (OVs) armed with GM-CSF lead to localized cytokine expression within the tumor microenvironment while enhancing tumor cell susceptibility to viral infection by driving these cells into the cell cycle. This effectively converts “cold” tumors characterized by low immune activity into “hot” tumors exhibiting high levels of immune activity (69). Additionally, GM-CSF-armed OVs promote DC recruitment and maturation at tumor sites, which enhances T-cell priming and generates robust anti-tumor immune responses (70). This process can also foster long-term immunological memory resulting in sustained anti-tumor effects. The first oncolytic HSV-1 armed with GM-CSF, talimogene laherparepvec (T-VEC), demonstrated significant anti-tumor efficacy leading to FDA and EMA approval for melanoma therapy (71–73). However, excessive GM-CSF release also aggravates the systemic symptoms, such as fatigue and elevated body temperature. More attention is paid to the inflammatory status of patients during treatment.

### 3.2 IL-12

To enhance the tumor resistance of NK cells and cytotoxic T lymphocytes, interleukin-12 (IL-12) was selected as a candidate gene for the insertion of oncolytic HSV-1. Its ability to reshape the tumor microenvironment while augmenting responses to checkpoint inhibitors underscores its therapeutic potential particularly when combined with other cancer immunotherapies establishing it as a formidable agent in anti-tumor immunity.

IL-12 facilitates CD4+ T-cell differentiation into Th1 cells that secrete elevated levels of interferon-gamma (IFN-γ), which subsequently activates NK cells and cytotoxic T lymphocytes (CTLs), thereby enhancing their anti-tumoral functions (74). Moreover IL-12 amplifies both growth rates and cytotoxic activities among NK cells alongside CD4+ and CD8+ T lymphocytes, resulting in increased production of perforin and granzyme B, which are key molecules essential for CTLs’ capacity to eradicate tumor cells (74, 75). Additionally, IL-12 promotes differentiation toward memory or effector T-cell phenotypes, thus improving precision persistence within targeting residual or metastatic malignant populations (76, 77).

Furthermore, IL-12 diminishes regulatory T-cell (Treg) and myeloid-derived suppressor cell (MDSC) populations within tumoral environments alleviating suppression mechanisms detrimental toward effective antitumoral responses (5, 78). It also drives macrophage polarization toward an M1 phenotype, a state characterized by pro-inflammatory properties conducive for inducing tumoricidal activity. By downregulating vascular endothelial growth factor (VEGF), IL-12 effectively reduces angiogenesis associated with tumors (25, 79).

Moreover, IL-12 sensitizes neoplasms toward checkpoint inhibitors like PD-1/PD-L1 blockade, thereby amplifying therapeutic efficacy (24, 80). With regard to promotion of tumor antigen presentation, death induced through IL-12-stimulated effectors releases TAAs further stimulating adaptive immunity assisting remaining malignant targets recognized by activated T cells (76, 77).

## 4 Others

In addition to the aforementioned wide-ranging applications in genetic modification techniques, several strategies for genetic modification demonstrate significant potential for clinical application (**Table 1**). A category of genetically modified viruses has been developed to enhance viral replication and tumor-specific cytotoxicity. The IRS1 and TRS1 genes from human cytomegalovirus (HCMV) have been inserted into HSV-2 to improve protein synthesis and replication by inhibiting PKR kinase activity and autophagy, thereby facilitating robust viral protein production and survival within tumor cells (81).

**Table 1:** Genetic modification of the modified oHSV.

Aim	Target genes	Related oncolytic HSV-1	References
Enhance the potency of oncolytic viruses	HCMV IRS1	C132,C134	(81)
	HCMV TRS1	C130	(81)
	GADD34	NG34, NG34 ScFvPD-1	(82)
	MyD116	GD116	(83)
	GALV-GP R-	OncoVEX^GALV/CD^	(84, 85)
	Nestin γ134.5	rQNestin34.5	(86, 87)
	angiostatin complementary	G47Δ-mAngio	(88)
Enhance the host immune response against the tumor	EphA2	C172, C170	(89)
	Flt3L	ONCR-177, G47Δ-Flt3L	(72, 90)
	IL-15	VG161	(91)
	anti-CTLA4	ONCR-177, RP2	(72)
Immunorecruitment and chemotactic infiltration	CCL2	M010,	(92)
	CCL4	ONCR-177	(72)
	CCL5	OV-Cmab-CCL5	(93)
Cooperate with PD-1 inhibitor	anti-PD-1 Fab	T3011	
	PD-L1B	VG161	(91)
	hPD-1scFv	YST-OVH, NG34 ScFvPD-1	(94, 95)
Prodrug invertase	cytochrome P450 enzyme	rRp450	(96)
	Fcy::Fur	OncoVEX^GALV/CD^	(85)
Light-activated cytotoxicity	KR	G47Δ-KR	(97)
Anti-inflammatory	IL-4	R8306	(98)
Weaken the replication and re-transmission	UL55	HF10/C-REV	(99)
	UL56		
	US3	R7041, MG18L	(100, 101)
	UL23	Dlsptk	(102)
Reduce immune escape	UL43	HF10/C-REV	(99)
	UL49.5		
	LAT		

HCMV, human cytomegalovirus; GADD34, growth arrest and DNA damage gene 34; MyD116, mouse myeloid differentiation protein 116; GALV-GP R−, gibbon ape leukemia virus membrane R− glycoprotein; EphA2, ephrin type-A receptor 2; Flt3L, Fms-related tyrosine kinase 3 ligand; IL-15, interleukin-15; anti-CTLA4, anti-cytotoxic T lymphocyte-associated protein 4; CCL2, chemokine (C–C motif) ligand 2; CCL4, chemokine (C–C motif) ligand 4; CCL5, chemokine (C–C motif) ligand 5; Fcy::Fur, yeast cytosine deaminase/uracil phospho-ribosyltransferase fusion; KR, KillerRed; IL-4, interleukin-4; LAT, linker for activation of T cells; PD-L1B, programmed death-ligand 1 B; hPD-1scFv, humanized single-chain variable fragment against human PD-1; UL55, UL56, UL23, UL43, UL49.5, unique long region 55, 56, 23, etc.; US3, unique short region 3. Gray indicates the distinct objectives, pink denotes the inserted genes, and blue signifies the representative strains harboring different types of inserted genes. Green marks the knockout genes, while yellow highlights the representative strains with various knockout genes.

GADD34 is homologous to γ134.5; like MyD116, it can substitute for γ134.5 to restore viral replication in glioblastoma and breast cancer cells, enhancing selective cytotoxicity (82, 83). The Gibbon leukemia virus fusion glycoprotein (GALV-GP) increases the efficiency of viral vector entry while inducing cell fusion, significantly boosting tumor cell death *in vitro* and promoting tumor shrinkage *in vivo (* 84, 85). The Nestin promoter drives selective replication in glioma cells, enhancing glioma suppression when combined with cyclophosphamide (86, 87).

To induce and facilitate host immune responses against tumors, numerous attempts have been made to insert various genes into oncolytic viruses (OVs). EphA2 induces anti-tumor immunity by generating EphA2-specific CD8+ T cells that are effective against resistant tumors (89). Flt3L promotes dendritic cell development, thereby enhancing both local and systemic anti-tumor immune responses (90). IL-15 amplifies NK cell and CD8+ T-cell responses while enhancing tumor-specific immune cycles as demonstrated in pancreatic cancer models (91). Anti-CTLA4 antibody ONCR-177 increases the CD8+ T-cell response specific to tumor antigens, effectively inhibiting metastatic tumors while bolstering memory responses (103).

Some studies focus on immunorecruitment and chemotactic infiltration of immune cells into tumors to improve the efficacy of oncolytic viruses against malignancies. Chemokine genes such as CCL2, CCL4, and CCL5 are incorporated into OVs to enhance immune cell infiltration within tumors. For instance, a γ134.5-deficient HSV-1 expressing CCL2 along with IL-12 enhances glioma killing capabilities (92), whereas OV-CIMab-CCL5 improves outcomes in glioblastoma patients (93).

Certain genetic engineering studies target synergy with immune checkpoint inhibitors for enhanced anti-tumor effects. PD-1, associated synergistic genes inserted into the HSV genome, include single, stranded variable fragment PD-1 (ScFvPD-1), variable region components of antibodies targeting programmed death receptor one (anti-PD-1 Fab),and portions acting as PD -1 blockers (PD-L1B). Incorporating ScFvPD-1 sequences into NG34 virus augments anti-tumoral responses prolonging survival rates observed across ovarian carcinoma models alongside those exhibiting glioblastomas, demonstrating synergistic benefits when paired with PI3K inhibitors (94, 104). The ScFvPD-1 gene is also integrated within YST-OVH aiming at promoting systemic antitumoral reactions through CTLA–4 or TIM–3 blockade (95).

Another broad category concerning genetic modifications applied toward OV focuses upon prodrug activation mechanisms. Infected tumoral environments allow the synthesis of prodrug invertase produced intracellularly via virally encoded proteins, converting non-toxic precursors and directly transforming them into therapeutic agents. As early as 1998, cytochrome P450 was introduced within HSV-I, enabling conversion processes whereby cyclophosphamide becomes activated specifically inside malignant tissues, leading toward notable anticancer effects evidenced across medulloblastoma atypical teratoid/rhabdomyosarcoma brain neoplasms among others (105, 106). This approach yielded substantial advantages during treatment regimens involving diverse oncological conditions including but not limited to those previously mentioned (96, 107, 108).

An additional strategy involves inserting a gene-encoding yeast cytosine deaminase/uracil phospho-ribosyltransferase fusion(Fcy::Fur) into HSV-I, prompting infected neoplastic entities capable of synthesizing said construct. Fcy::Fur fusion catalyzes transformation processes wherein five-fluorocytosine (5-FC) is converted selectively, yielding toxic derivatives known as five-fluorouracil (5-FU), effectively targeting only malignant cellular populations without adversely affecting surrounding healthy tissue structures (85).

Recently, Kazuhide’s team successfully integrated killer red (KR) gene allowing light-induced singlet oxygen generation, which markedly enhanced overall effectiveness regarding treatments administered under laser irradiation particularly noted among cases involving both gliobastomatosis multiple myelomas (97).

To enhance safety profiles related specifically toward employing HSV-1-based therapeutics aimed at combating cancers, certain critical genomic deletions occur preventing uncontrolled propagation/infection events. Two primary methodologies exist focusing upon limiting risks tied closely together utilizing these engineered strains. One method entails restricting replicative capacity particle assembly through deletion, such as UL55, UL56, US3, and UL23, thus confining resultant virulence strictly localized around affected sites (99, 102, 109). Another tactic employs removing particular loci inclusive of UL43, UL49.5, and LAT, mitigating escape routes available and henceforth increasing the likelihood of successful elimination efforts directed toward residual pathogenic threats encountered post-treatment interventions (99).

## 5 Clinical trials

Preclinical studies have identified a substantial number of oHSVs with diverse antitumor properties. To gain a deeper understanding of the clinical application of oHSVs, we conducted a review of 34 published oHSV clinical trials spanning the past two decades (**Table 2**). Over half of these clinical trials were concentrated in Phases I and II, comprising 67% of the total. The three most common treatment methods were the injection of T-VEC, the combination of T-VEC injection and pembrolizumab, and the injection of G47Δ, which accounted for 26%, 11%, and 5.9% of the total, respectively. Among the tumors targeted by oHSV clinical trials, the top 2 were melanoma and brain tumors, representing 50% and 17.6% of the total, respectively. The oHSV type most frequently reported in clinical trials was T-VEC (n=22), accounting for 64% of all clinical trials. Notably, 22 out of the 34 clinical trials were conducted in the past 5 years, indicating a significant increase in research interest in this field.

**Table 2:** Published clinical trials with oHSV.

Year (published)	Phase	oHSV applied	Method	Tumor	References
2024	Phase IB	orienx010	orienx010+anti-PD-1	Toripalimab melanoma	(110)
2024	Phase II	T-VEC	T-VEC+radiotherapy	cutaneous metastases from solid tumors	(111)
2024	Phase II	T-VEC	T-VEC+pembrolizumab	melanoma	(112)
2023	Phase I	CAN-3110	CAN-3110	glioblastoma	(113)
2023	Phase II	T-VEC	T-VEC+surgery	melanoma	(114)
2023	Phase II	T-VEC	T-VEC+ipilimumab	melanoma	(115)
2022	Phase I	T-VEC	T-VEC+CD1c (BDCA-1)+ +/− CD141 (BDCA-3)+ myDCs	melanoma	(116)
2022	Phase III	T-VEC	T-VEC+pembrolizumab	melanoma	(117)
2022	Phase II	G47Δ	G47Δ	glioblastoma	(118)
2022	Phase I/II	G47Δ	G47Δ	glioblastoma	(119)
2022	Phase I	T-VEC	T-VEC	melanoma	(120)
2022	Phase IB	orienx010	orienx010	melanoma	(73)
2021	Phase II	T-VEC	T-VEC	breast cancer	(121)
2021	Phase II	T-VEC	T-VEC+surgery	melanoma	(122)
2021	Phase IB/II	T-VEC	T-VEC+external beam radiation therapy	sarcoma	(123)
2021	Phase II	T-VEC	T-VEC	melanoma	(124)
2021	Phase I	T-VEC	T-VEC+neoadjuvant chemotherapy	breast cancer	(125)
2020	Phase IB	T-VEC	T-VEC+pembrolizumab	head and neck squamous cell carcinoma	(126)
2020	Phase II	T-VEC	T-VEC+pembrolizumab	sarcoma	(127)
2019	Phase I	HSV1716	HSV1716	relapsed or refractory extra-cranial solid cancers	(128)
2019	Phase II	T-VEC	T-VEC	melanoma	(129)
2019	Phase III	T-VEC	T-VEC	melanoma	(130)
2018	Phase I	HF10	HF10+erlotinib and gemcitabine	pancreatic cancer	(131)
2018	Phase II	T-VEC	T-VEC+ipilimumab	melanoma	(132)
2017	Phase I	G207	G207	malignant brain tumors	(133)
2016	Phase III	T-VEC	T-VEC	melanoma	(134)
2016	Phase I	M032	M032	malignant brain tumors	(135)
2015	Phase III	T-VEC	T-VEC	melanoma	(136)
2014	Phase I	HF10	HF10	refractory superficial solid tumors	(137)
2014	Phase I	G207	G207+radiation	malignant brain tumors	(138)
2010	Phase I/II	T-VEC	T-VEC+chemoradiotherapy	head and neck squamous cell carcinoma	(139)
2010	Phase III	T-VEC	T-VEC	melanoma	(140)
2009	Phase II	T-VEC	T-VEC	melanoma	(141)
2006	Phase I	NV1020	NV1020	hepatic colorectal metastases	(142)

## 6 Discussion

In this review, we observed that the majority of oHSV clinical trials have employed various forms of viral modifications, such as deletions of genes γ134.5, US11, US12, and UL39, or the expression of transgenes like GM-CSF and IL-12. We also explored various gene modifications, which, despite not having been evaluated in clinical trials, represent a promising direction for future oncolytic virus research. Although oncolytic virotherapy is a promising anti-tumor technique, it is still facing several challenges.

The effectiveness of oncolytic viruses (OVs) is modest despite good safety. Viral genetic engineering improvements may enhance efficacy, but there are still obstacles in clinical trials, like balancing viral replication and immune responses, optimizing delivery routes, and achieving tumor-specific targeting.

During oncolytic virotherapy, it is imperative to achieve equilibrium between viral proliferation and the host’s anti-viral immune response. The ideal immune response is to allow viral replication early in oncolytic virotherapy and to initiate humoral immunity and clear the virus quickly at the end of treatment. The host immune system is crucial for tumor elimination but can clear OVs prematurely, limiting their therapeutic potential. Optimizing virus delivery and suppressing early immune responses give the virus more time for anti-tumor action. One of the strategies currently ongoing is to optimize delivery methods so that the virus moves silently into tumor cells before the host generates an immune response to clear the virus. Another strategy is to suppress the host immune response early on treatment, thereby improving the infection efficiency of the oncolytic virus. Upon completion of therapy, the introduction of antiviral medications expedites the virus’ elimination (143).

The current delivery methods include intratumoral injection and intravenous delivery. Intratumoral injection has the limitation of accessible tumors and is practically difficult in deep-seated or metastatic cases. For inaccessible tumors, imaging-guided or surgical approaches are required, which further complicate intratumoral injection. Intravenous delivery is more convenient than intratumoral injection. However, it requires high specificity to target tumors effectively, not to mention that it has risks of systemic toxicity and immune clearance.

Moreover, OVs as monotherapy may not achieve best therapeutic results. OVs are usually combined with other therapies, including immune checkpoint blockade or traditional anti-tumor therapies, to increase efficacy. Recently, integrating OVs with chimeric antigen receptor (CAR)-T cell therapy has emerged as an option. It could facilitate targeted delivery while improving bioavailability and enhancing tumor specificity. Furthermore, optimizing timing and dosing remains crucial for maximizing synergy between OVs and CAR-T cells (144, 145). A comprehensive regimen combining stereotactic body radiotherapy, oncolytic virotherapy, and pembrolizumab was used in clinical studies of metastatic non-small-cell lung cancer. The results demonstrate the superior prognosis of the comprehensive treatment regimen over conventional chemotherapy and pembrolizumab alone (146).

Potential safety issues of oncolytic virus therapy have also been suggested in clinical trials. For example, tumor cells died in large numbers after virus injection, resulting in the release of large amounts of antigenic material and cytokines. If the above-mentioned process occurs in a short time, it can lead to the life-threatening cytokine release syndrome. In addition, after the death of tumor cells, intracellular substances enter the circulation system and affect the coagulation system, which can lead to thrombosis or bleeding events. In addition, viruses may also cause insertional mutagenesis in host cells; for instance, the oncolytic adenovirus-based studies have found out the integration of viral genes into the host genome. As a kind of DNA virus, the possibility of insertional mutagenesis of HSV-1 virus is relatively small in theory, while long-term observation and studies are also needed toward this issue (147). Oncolytic HSV-1 has the potential to move through blood–brain barrier and infect the central nervous system, which, on the one hand, makes this type of oncolytic virus a candidate for the treatment of neurogenic malignancies, and on the other hand, increase the risk of central nervous system virus infection during the treatment of other tumors. Genetic modification is commonly used as one of the preventive strategies to reduce the pathogenicity of oncolytic viruses and improve their specificity for tumor cells. For example, G47Δ silenced γ134.5, UL39, US12, and US11 genes simultaneously (118). Clinical trials have shown that this kind of virus can barely replicate *in vivo*; therefore, treatment with the right dose of injected virus can safely treat tumors. Another preventive strategy is to combine oncolytic virus therapy with tumor immune checkpoint therapy or chemotherapy to kill the tumor while reducing the amount of oncolytic virus injection during the treatment. This strategy is currently widely used in clinical trial, such as the use of T-VEC virus strain combined with anti-PD-1 treatment (112, 126, 127).

As an increasing number of clinical trials explore newly engineered oncolytic virotherapies, these advancements are poised to yield significant breakthroughs in related research and promote the widespread adoption of oncolytic virotherapy for cancer treatment.

## References

[1] Santos AJ Lima DSGV Cordeiro SM Silva LM Silva SJ Rocha PS. Oncolytic virus therapy in cancer: A current review. World J Virol. (2021) 10:229–55. doi: 10.5501/wjv.v10.i5.229 PMC8474975 34631474

[2] Broberg EK Hukkanen V. Immune response to herpes simplex virus and gamma134.5 deleted HSV vectors. Curr Gene Ther. (2005) 5:523–30. doi: 10.2174/156652305774329267 16250892

[3] Fukuhara H Takeshima Y Todo T. Triple-mutated oncolytic herpes virus for treating both fast- and slow-growing tumors. Cancer Sci. (2021) 112:3293–301. doi: 10.1111/cas.14981 PMC8353919 34036669

[4] Kumar A Taghi Khani A Sanchez Ortiz A Swaminathan S. GM-CSF: A double-edged sword in cancer immunotherapy. Front Immunol. (2022) 13:901277. doi: 10.3389/fimmu.2022.901277 35865534 PMC9294178

[5] Agliardi G Liuzzi AR Hotblack A De Feo D Nunez N Stowe CL. Intratumoral IL-12 delivery empowers CAR-T cell immunotherapy in a pre-clinical model of glioblastoma. Nat Commun. (2021) 12:444. doi: 10.1038/s41467-020-20599-x 33469002 PMC7815781

[6] Schwarz E Carson WE. Analysis of potential biomarkers of response to IL-12 therapy. J Leukocyte Biol. (2022) 112:557–67. doi: 10.1002/JLB.5RU1221-675R PMC9542878 35790025

[7] Kobiler O Afriat A. The fate of incoming HSV-1 genomes entering the nucleus. Curr Issues Mol Biol. (2021) 41:221–66. doi: 10.21775/cimb.041.221 32879055

[8] Boldogkoi Z Szucs A Balazs Z Sharon D Snyder M Tombacz D. Transcriptomic study of Herpes simplex virus type-1 using full-length sequencing techniques. Sci Data. (2018) 5:180266. doi: 10.1038/sdata.2018.266 30480662 PMC6257044

[9] Goins WF Wolfe D Krisky DM Bai Q Burton EA Fink DJ. Delivery using herpes simplex virus: an overview. Methods Mol Biol. (2004) 246:257–99. doi: 10.1385/1-59259-650-9:257 14970599

[10] Carroll NM Chase M Chiocca EA Tanabe KK. The effect of ganciclovir on herpes simplex virus-mediated oncolysis. J Surg Res. (1997) 69:413–7. doi: 10.1006/jsre.1997.5089 9224416

[11] Ripa I Andreu S Lopez-Guerrero JA Bello-Morales R. Interplay between autophagy and herpes simplex virus type 1: ICP34.5, one of the main actors. Int J Mol Sci. (2022) 23:13643. doi: 10.3390/ijms232113643 PMC9655901 36362429

[12] Mao H Rosenthal KS. Strain-dependent structural variants of herpes simplex virus type 1 ICP34.5 determine viral plaque size, efficiency of glycoprotein processing, and viral release and neuroinvasive disease potential. J Virol. (2003) 77:3409–17. doi: 10.1128/jvi.77.6.3409-3417.2003 PMC149531 12610116

[13] Cassady KA Gross M Roizman B. The herpes simplex virus US11 protein effectively compensates for the gamma1(34.5) gene if present before activation of protein kinase R by precluding its phosphorylation and that of the alpha subunit of eukaryotic translation initiation factor 2. J Virol. (1998) 72:8620–6. doi: 10.1128/JVI.72.11.8620-8626.1998 PMC110273 9765401

[14] Ahn K Meyer TH Uebel S Sempe P Djaballah H Yang Y. Molecular mechanism and species specificity of TAP inhibition by herpes simplex virus ICP47. EMBO J. (1996) 15:3247–55. doi: 10.1002/j.1460-2075.1996.tb00689.x PMC451885 8670825

[15] Goldsmith K Chen W Johnson DC Hendricks RL. Infected cell protein (ICP)47 enhances herpes simplex virus neurovirulence by blocking the CD8+ T cell response. J Exp Med. (1998) 187:341–8. doi: 10.1084/jem.187.3.341 PMC2212130 9449714

[16] Bommareddy PK Patel A Hossain S Kaufman HL. Talimogene laherparepvec (T-VEC) and other oncolytic viruses for the treatment of melanoma. Am J Clin Dermatol. (2017) 18:1–15. doi: 10.1007/s40257-016-0238-9 27988837 PMC8977104

[17] Kanai R Zaupa C Sgubin D Antoszczyk SJ Martuza RL Wakimoto H. Effect of γ34.5 deletions on oncolytic herpes simplex virus activity in brain tumors. J Virol. (2012) 86:4420–31. doi: 10.1128/JVI.00017-12 PMC3318611 22345479

[18] Pan C Cai Q Li X Li L Yang L Chen Y. Correction to: Enhancing the HSV-1-mediated antitumor immune response by suppressing Bach1. Cell Mol Immunol. (2022) 19:754. doi: 10.1038/s41423-022-00860-7 35562553 PMC9151714

[19] Charron AJ Ward SL North BJ Ceron S Leib DA. The US11 gene of herpes simplex virus 1 promotes neuroinvasion and periocular replication following corneal infection. J Virol. (2019) 93:e02246–18. doi: 10.1128/JVI.02246-18 PMC6475787 30760571

[20] Chowdhury FZ Ramos HJ Davis LS Forman J Farrar JD. IL-12 selectively programs effector pathways that are stably expressed in human CD8+ effector memory T cells. vivo. Blood. (2011) 118:3890–900. doi: 10.1182/blood-2011-05-357111 PMC3193266 21832277

[21] Kim K Moon D Kong SJ Lee YS Yoo Y Kim S. Antitumor effects of IL-12 and GM-CSF co-expressed in an engineered oncolytic HSV-1. Gene Ther. (2021) 28:186–98. doi: 10.1038/s41434-020-00205-x 33149278

[22] Ma W He H Wang H. Oncolytic herpes simplex virus and immunotherapy. BMC Immunol. (2018) 19:40. doi: 10.1186/s12865-018-0281-9 30563466 PMC6299639

[23] Lellahi SM Azeem W Hua Y Gabriel B Paulsen Rye K Reikvam H. GM-CSF, Flt3-L and IL-4 affect viability and function of conventional dendritic cell types 1 and 2. Front Immunol. (2023) 13:1058963. doi: 10.3389/fimmu.2022.1058963 36713392 PMC9880532

[24] Minnar CM Chariou PL Horn LA Hicks KC Palena C Schlom J. Tumor-targeted interleukin-12 synergizes with entinostat to overcome PD-1/PD-L1 blockade-resistant tumors harboring MHC-I and APM deficiencies. J Immunother Cancer. (2022) 10:e004561. doi: 10.1136/jitc-2022-004561 PMC9240938 35764364

[25] Bonfanti A Lissoni P Bucovec R Rovelli F Brivio F Fumagalli L. Changes in circulating dendritic cells and IL-12 in relation to the angiogenic factor VEGF during IL-2 immunotherapy of metastatic renal cell cancer. Int J Biol Markers. (2000) 15:161–4. doi: 10.1177/172460080001500206 10883890

[26] Chou J Kern ER Whitley RJ Roizman B. Mapping of herpes simplex virus-1 neurovirulence to gamma 134.5, a gene nonessential for growth in culture. Science. (1990) 250:1262–6. doi: 10.1126/science.2173860 2173860

[27] Zhang C Tang J Xie J Zhang H Li Y Zhang J. A conserved domain of herpes simplex virus ICP34.5 regulates protein phosphatase complex in mammalian cells. FEBS Lett. (2008) 582:171–6. doi: 10.1016/j.febslet.2007.11.082 18068675

[28] Cheng G Gross M Brett M He B. AlaArg motif in the carboxyl terminus of the γ_1_ 34.5 protein of herpes simplex virus type 1 is required for the formation of a high-molecular-weight complex that dephosphorylates eIF-2α. J Virol. (2001) 75:3666–74. doi: 10.1128/JVI.75.8.3666-3674.2001 PMC114858 11264356

[29] He B Gross M Roizman B. The gamma(1)34.5 protein of herpes simplex virus 1 complexes with protein phosphatase 1alpha to dephosphorylate the alpha subunit of the eukaryotic translation initiation factor 2 and preclude the shutoff of protein synthesis by double-stranded RNA-activated protein kinase. P Natl Acad Sci USA. (1997) 94:843–8. doi: 10.1073/pnas.94.3.843 PMC19601 9023344

[30] He B Gross M Roizman B. The gamma134.5 protein of herpes simplex virus 1 has the structural and functional attributes of a protein phosphatase 1 regulatory subunit and is present in a high molecular weight complex with the enzyme in infected cells. J Biol Chem. (1998) 273:20737–43. doi: 10.1074/jbc.273.33.20737 9694816

[31] Li Y Zhang C Chen X Yu J Wang Y Yang Y. ICP34.5 protein of herpes simplex virus facilitates the initiation of protein translation by bridging eukaryotic initiation factor 2alpha (eIF2alpha) and protein phosphatase 1. J Biol Chem. (2011) 286:24785–92. doi: 10.1074/jbc.M111.232439 PMC3137054 21622569

[32] Harland J Dunn P Cameron E Conner J Brown SM. The herpes simplex virus (HSV) protein ICP34.5 is a virion component that forms a DNA-binding complex with proliferating cell nuclear antigen and HSV replication proteins. J Neurovirol. (2003) 9:477–88. doi: 10.1080/13550280390218788 12907392

[33] Liu X Ma Y Voss K van Gent M Chan YK Gack MU. The herpesvirus accessory protein gamma134.5 facilitates viral replication by disabling mitochondrial translocation of RIG-I. PLoS Pathog. (2021) 17:e1009446. doi: 10.1371/journal.ppat.1009446 33770145 PMC7996975

[34] Pan S Liu X Ma Y Cao Y He B. Herpes simplex virus 1 gamma(1)34.5 protein inhibits STING activation that restricts viral replication. J Virol. (2018) 92:e01015–18. doi: 10.1128/JVI.01015-18 PMC6158424 30045990

[35] Orvedahl A Alexander D Talloczy Z Sun Q Wei Y Zhang W. HSV-1 ICP34.5 confers neurovirulence by targeting the Beclin 1 autophagy protein. Cell Host Microbe. (2007) 1:23–35. doi: 10.1016/j.chom.2006.12.001 18005679

[36] Leib DA Alexander DE Cox D Yin J Ferguson TA. Interaction of ICP34.5 with Beclin 1 modulates herpes simplex virus type 1 pathogenesis through control of CD4+ T-cell responses. J Virol. (2009) 83:12164–71. doi: 10.1128/JVI.01676-09 PMC2786728 19759141

[37] Gobeil PAM Leib DA. Herpes simplex virus gamma34.5 interferes with autophagosome maturation and antigen presentation in dendritic cells. Mbio. (2012) 3:e212–67. doi: 10.1128/mBio.00267-12 PMC3470650 23073763

[38] Jin H Yan Z Ma Y Cao Y He B. A herpesvirus virulence factor inhibits dendritic cell maturation through protein phosphatase 1 and Ikappa B kinase. J Virol. (2011) 85:3397–407. doi: 10.1128/JVI.02373-10 PMC3067875 21248029

[39] Li Q Zheng Z Liu Y Zhang Z Liu Q Meng J. 2C proteins of enteroviruses suppress IKKbeta phosphorylation by recruiting protein phosphatase 1. J Virol. (2016) 90:5141–51. doi: 10.1128/JVI.03021-15 PMC4859720 26962213

[40] McGeoch DJ Dolan A Donald S Rixon FJ. Sequence determination and genetic content of the short unique region in the genome of herpes simplex virus type 1. J Mol Biol. (1985) 181:1–13. doi: 10.1016/0022-2836(85)90320-1 2984429

[41] Johnson PA MacLean C Marsden HS Dalziel RG Everett RD. The product of gene US11 of herpes simplex virus type 1 is expressed as a true late gene. J Gen Virol. (1986) 67:871–83. doi: 10.1099/0022-1317-67-5-871 3009688

[42] Khoo D Perez C Mohr I. Characterization of RNA determinants recognized by the arginine- and proline-rich region of Us11, a herpes simplex virus type 1-encoded double-stranded RNA binding protein that prevents PKR activation. J Virol. (2002) 76:11971–81. doi: 10.1128/jvi.76.23.11971-11981.2002 PMC136894 12414939

[43] Poppers J Mulvey M Khoo D Mohr I. Inhibition of PKR activation by the proline-rich RNA binding domain of the herpes simplex virus type 1 Us11 protein. J Virol. (2000) 74:11215–21. doi: 10.1128/jvi.74.23.11215-11221.2000 PMC113216 11070019

[44] Xing J Wang S Lin R Mossman KL Zheng C. Herpes simplex virus 1 tegument protein US11 downmodulates the RLR signaling pathway via direct interaction with RIG-I and MDA-5. J Virol. (2012) 86:3528–40. doi: 10.1128/JVI.06713-11 PMC3302539 22301138

[45] Kew C Lui P Chan C Liu X Au SWN Mohr I. Suppression of PACT-induced type I interferon production by herpes simplex virus 1 Us11 protein. J Virol. (2013) 87:13141–9. doi: 10.1128/JVI.02564-13 PMC3838286 24067967

[46] Sanchez R Mohr I. Inhibition of cellular 2’-5’ oligoadenylate synthetase by the herpes simplex virus type 1 Us11 protein. J Virol. (2007) 81:3455–64. doi: 10.1128/JVI.02520-06 PMC1866071 17229694

[47] Pierantoni GM Fedele M Pentimalli F Benvenuto G Pero R Viglietto G. High mobility group I (Y) proteins bind HIPK2, a serine-threonine kinase protein which inhibits cell growth. Oncogene. (2001) 20:6132–41. doi: 10.1038/sj.onc.1204635 11593421

[48] Giraud S Diaz-Latoud C Hacot S Textoris J Bourette RP Diaz J. US11 of herpes simplex virus type 1 interacts with HIPK2 and antagonizes HIPK2-induced cell growth arrest. J Virol. (2004) 78:2984–93. doi: 10.1128/jvi.78.6.2984-2993.2004 PMC353731 14990717

[49] Aubert M Krantz EM Jerome KR. Herpes simplex virus genes Us3, Us5, and Us12 differentially regulate cytotoxic T lymphocyte-induced cytotoxicity. Viral Immunol. (2006) 19:391–408. doi: 10.1089/vim.2006.19.391 16987059

[50] Whitley RJ Roizman B. Herpes simplex virus infections. Lancet. (2001) 357:1513–8. doi: 10.1016/S0140-6736(00)04638-9 11377626

[51] He B Chou J Brandimarti R Mohr I Gluzman Y Roizman B. Suppression of the phenotype of gamma(1)34.5- herpes simplex virus 1: failure of activated RNA-dependent protein kinase to shut off protein synthesis is associated with a deletion in the domain of the alpha47 gene. J Virol. (1997) 71:6049–54. doi: 10.1128/JVI.71.8.6049-6054.1997 PMC191863 9223497

[52] Cassady KA Gross M Gillespie GY Roizman B. Second-site mutation outside of the U(S)10-12 domain of Deltagamma(1)34.5 herpes simplex virus 1 recombinant blocks the shutoff of protein synthesis induced by activated protein kinase R and partially restores neurovirulence. J Virol. (2002) 76:942–9. doi: 10.1128/jvi.76.3.942-949.2002 PMC135782 11773369

[53] Mocarski ES Guo H Kaiser WJ. Necroptosis: The Trojan horse in cell autonomous antiviral host defense. Virology. (2015) 479-480:160–6. doi: 10.1016/j.virol.2015.03.016 PMC5115625 25819165

[54] Shanmugam N Baker MODG Sanz-Hernandez M Sierecki E Gambin Y Steain M. Herpes simplex virus encoded ICP6 protein forms functional amyloid assemblies with necroptosis-associated host proteins. Biophys Chem. (2021) 269:106524. doi: 10.1016/j.bpc.2020.106524 33348174

[55] Yamamoto S Deckter LA Kasai K Chiocca EA Saeki Y. Imaging immediate-early and strict-late promoter activity during oncolytic herpes simplex virus type 1 infection and replication in tumors. Gene Ther. (2006) 13:1731–6. doi: 10.1038/sj.gt.3302831 16871231

[56] Aldrak N Alsaab S Algethami A Bhere D Wakimoto H Shah K. Oncolytic herpes simplex virus-based therapies for cancer. Cells-Basel. (2021) 10:1541. doi: 10.3390/cells10061541 PMC8235327 34207386

[57] Nakamura H Kasuya H Mullen JT Yoon SS Pawlik TM Chandrasekhar S. Regulation of herpes simplex virus gamma(1)34.5 expression and oncolysis of diffuse liver metastases by Myb34.5. J Clin Invest. (2002) 109:871–82. doi: 10.1172/JCI10623 PMC150923 11927614

[58] Hu H Wu G Shu Z Yu D Nan N Yuan F. ICP6 prevents RIP1 activation to hinder necroptosis signaling. Front Cell Dev Biol. (2020) 8:595253. doi: 10.3389/fcell.2020.595253 33195272 PMC7661466

[59] Katsura T Iwai S Ota Y Shimizu H Ikuta K Yura Y. The effects of trichostatin A on the oncolytic ability of herpes simplex virus for oral squamous cell carcinoma cells. Cancer Gene Ther. (2009) 16:237–45. doi: 10.1038/cgt.2008.81 18949013

[60] Yokota Y Inoue H Matsumura Y Nabeta H Narusawa M Watanabe A. Absence of LTB4/BLT1 axis facilitates generation of mouse GM-CSF–induced long-lasting antitumor immunologic memory by enhancing innate and adaptive immune systems. Blood. (2012) 120:3444–54. doi: 10.1182/blood-2011-10-383240 22936657

[61] Katz S Zsiros V Dóczi N Szabó A Biczó Á Kiss AL. GM-CSF and GM-CSF receptor have regulatory role in transforming rat mesenteric mesothelial cells into macrophage-like cells. Inflammation Res. (2016) 65:827–36. doi: 10.1007/s00011-016-0967-5 27364613

[62] Xiang X Ma H Chen Y Zhang D Ma S Wang H. GM-CSF-miRNA-jak2/stat3 signaling mediates chemotherapy-induced cancer cell stemness in gastric cancer. Front Pharmacol. (2022) 13:855351. doi: 10.3389/fphar.2022.855351 35600882 PMC9117965

[63] Shao S Chen C Shi G Zhou Y Wei Y Wu L. JAK inhibition ameliorated experimental autoimmune encephalomyelitis by blocking GM-CSF-driven inflammatory signature of monocytes. Acta Pharm Sin B. (2023) 13:4185–201. doi: 10.1016/j.apsb.2023.07.026 PMC10547959 37799385

[64] Liu L Cheng Y Zhang F Chen J Tian P Shi W. IL-2/GM-CSF enhances CXCR3 expression in CAR-T cells via the PI3K/AKT and ERK1/2 pathways. J Cancer Res Clin. (2023) 149:5547–57. doi: 10.1007/s00432-022-04509-w PMC11798273 36474002

[65] Petrina M Martin J Basta S. Granulocyte macrophage colony-stimulating factor has come of age: From a vaccine adjuvant to antiviral immunotherapy. Cytokine Growth F R. (2021) 59:101–10. doi: 10.1016/j.cytogfr.2021.01.001 PMC8064670 33593661

[66] Achuthan AA Lee K Hamilton JA. Targeting GM-CSF in inflammatory and autoimmune disorders. Semin Immunol. (2021) 54:101523. doi: 10.1016/j.smim.2021.101523 34776300

[67] Jaguin M Houlbert N Fardel O Lecureur V. Polarization profiles of human M-CSF-generated macrophages and comparison of M1-markers in classically activated macrophages from GM-CSF and M-CSF origin. Cell Immunol. (2013) 281:51–61. doi: 10.1016/j.cellimm.2013.01.010 23454681

[68] Helft J Böttcher J Chakravarty P Zelenay S Huotari J Schraml BU. GM-CSF mouse bone marrow cultures comprise a heterogeneous population of CD11c+MHCII+ Macrophages and dendritic cells. Immunity. (2015) 42:1197–211. doi: 10.1016/j.immuni.2015.05.018 26084029

[69] DeRubertis BG Stiles BM Bhargava A Gusani NJ Hezel M D’Angelica M. Cytokine-secreting herpes viral mutants effectively treat tumor in a murine metastatic colorectal liver model by oncolytic and T-cell-dependent mechanisms. Cancer Gene Ther. (2007) 14:590–7. doi: 10.1038/sj.cgt.7701053 17431402

[70] Che Y Chong C Limova M Morris L Reddy SA Chang A. Resolution of metastatic neck nodes associated with a periauricular cutaneous squamous cell carcinoma after intranodal injection of talimogene laherparepvec. Jaad Case Rep. (2024) 46:92–4. doi: 10.1016/j.jdcr.2024.02.008 PMC10992271 38577494

[71] Liu BL Robinson M Han Z Branston RH English C Reay P. ICP34.5 deleted herpes simplex virus with enhanced oncolytic, immune stimulating, and anti-tumour properties. Gene Ther. (2003) 10:292–303. doi: 10.1038/sj.gt.3301885 12595888

[72] Chaurasiya S Fong Y Warner SG. Oncolytic virotherapy for cancer: clinical experience. Biomedicines. (2021) 9:419. doi: 10.3390/biomedicines9040419 PMC8069290 33924556

[73] Cui C Wang X Lian B Ji Q Zhou L Chi Z. OrienX010, an oncolytic virus, in patients with unresectable stage IIIC-IV melanoma: a phase Ib study. J Immunother Cancer. (2022) 10:e004307. doi: 10.1136/jitc-2021-004307 PMC8984036 35383116

[74] Nguyen HM Guz-Montgomery K Saha D. Oncolytic virus encoding a master pro-inflammatory cytokine interleukin 12 in cancer immunotherapy. Cells-Basel. (2020) 9:400. doi: 10.3390/cells9020400 PMC7072539 32050597

[75] Kodama T Takeda K Shimozato O Hayakawa Y Atsuta M Kobayashi K. Perforin-dependent NK cell cytotoxicity is sufficient for anti-metastatic effect of IL-12. Eur J Immunol. (1999) 29:1390–6. doi: 10.1002/(SICI)1521-4141(199904)29:04<1390::AID-IMMU1390>3.0.CO;2-C 10229107

[76] Chang J Cho JH Lee SW Choi SY Ha SJ Sung YC. IL-12 priming during *in vitro* antigenic stimulation changes properties of CD8 T cells and increases generation of effector and memory cells. J Immunol. (2004) 172:2818–26. doi: 10.4049/jimmunol.172.5.2818 14978082

[77] Kilinc MO Aulakh KS Nair RE Jones SA Alard P Kosiewicz MM. Reversing tumor immune suppression with intratumoral IL-12: activation of tumor-associated T effector/memory cells, induction of T suppressor apoptosis, and infiltration of CD8+ T effectors. J Immunol. (2006) 177:6962–73. doi: 10.4049/jimmunol.177.10.6962 17082611

[78] Choi JN Sun EG Cho SH. IL-12 enhances immune response by modulation of myeloid derived suppressor cells in tumor microenvironment. Chonnam Med J. (2019) 55:31–9. doi: 10.4068/cmj.2019.55.1.31 PMC6351325 30740338

[79] Brivio F Lissoni P Rovelli F Nespoli A Uggeri F Fumagalli L. Effects of IL-2 preoperative immunotherapy on surgery-induced changes in angiogenic regulation and its prevention of VEGF increase and IL-12 decline. Hepatogastroenterology. (2002) 49:385–7. 11995457

[80] Garris CS Arlauckas SP Kohler RH Trefny MP Garren S Piot C. Successful anti-PD-1 cancer immunotherapy requires T cell-dendritic cell crosstalk involving the cytokines IFN-gamma and IL-12. Immunity. (2022) 55:1749. doi: 10.1016/j.immuni.2022.07.021 36103861

[81] Shah AC Parker JN Gillespie GY Lakeman FD Meleth S Markert JM. Enhanced antiglioma activity of chimeric HCMV/HSV-1 oncolytic viruses. Gene Ther. (2007) 14:1045–54. doi: 10.1038/sj.gt.3302942 17429445

[82] Nakashima H Nguyen T Kasai K Passaro C Ito H Goins WF. Toxicity and efficacy of a novel GADD34-expressing oncolytic HSV-1 for the treatment of experimental glioblastoma. Clin Cancer Res. (2018) 24:2574–84. doi: 10.1158/1078-0432.CCR-17-2954 PMC6800093 29511029

[83] Cheng L Jiang H Fan J Wang J Hu P Ruan Y. A novel oncolytic herpes simplex virus armed with the carboxyl-terminus of murine MyD116 has enhanced anti-tumour efficacy against human breast cancer cells. Oncol Lett. (2018) 15:7046–52. doi: 10.3892/ol.2018.8247 PMC5962873 29849789

[84] Price DL Lin S Han Z Simpson G Coffin RS Wong J. Oncolysis using herpes simplex virus type 1 engineered to express cytosine deaminase and a fusogenic glycoprotein for head and neck squamous cell carcinoma. Arch Otolaryngol Head Neck Surg. (2010) 136:151–8. doi: 10.1001/archoto.2009.214 PMC2824889 20157061

[85] Simpson GR Han Z Liu B Wang Y Campbell G Coffin RS. Combination of a fusogenic glycoprotein, prodrug activation, and oncolytic herpes simplex virus for enhanced local tumor control. Cancer Res. (2006) 66:4835–42. doi: 10.1158/0008-5472.CAN-05-4352 16651439

[86] Kambara H Okano H Chiocca EA Saeki Y. An oncolytic HSV-1 mutant expressing ICP34.5 under control of a nestin promoter increases survival of animals even when symptomatic from a brain tumor. Cancer Res. (2005) 65:2832–9. doi: 10.1158/0008-5472.CAN-04-3227 15805284

[87] Okemoto K Wagner B Meisen H Haseley A Kaur B Chiocca EA. STAT3 activation promotes oncolytic HSV1 replication in glioma cells. PLoS One. (2013) 8:e71932. doi: 10.1371/journal.pone.0071932 23936533 PMC3732216

[88] Zhang W Fulci G Buhrman JS Stemmer-Rachamimov AO Chen JW Wojtkiewicz GR. Bevacizumab with angiostatin-armed oHSV increases antiangiogenesis and decreases bevacizumab-induced invasion in U87 glioma. Mol Ther. (2012) 20:37–45. doi: 10.1038/mt.2011.187 21915104 PMC3255598

[89] Ghonime MG Saini U Kelly MC Roth JC Wang P Chen C. Eliciting an immune-mediated antitumor response through oncolytic herpes simplex virus-based shared antigen expression in tumors resistant to viroimmunotherapy. J Immunother Cancer. (2021) 9:e002939. doi: 10.1136/jitc-2021-002939 PMC8488720 34599026

[90] Barnard Z Wakimoto H Zaupa C Patel AP Klehm J Martuza RL. Expression of FMS-like tyrosine kinase 3 ligand by oncolytic herpes simplex virus type I prolongs survival in mice bearing established syngeneic intracranial Malignant glioma. Neurosurgery. (2012) 71:741–8, 748. doi: 10.1227/NEU.0b013e318260fd73 22653387 PMC3921689

[91] Shen Y Song W Lin D Zhang X Wang M Li Y. VG161 activates systemic antitumor immunity in pancreatic cancer models as a novel oncolytic herpesvirus expressing multiple immunomodulatory transgenes. J Med Virol. (2023) 95:e28108. doi: 10.1002/jmv.28108 36042555 PMC10087349

[92] Parker JN Meleth S Hughes KB Gillespie GY Whitley RJ Markert JM. Enhanced inhibition of syngeneic murine tumors by combinatorial therapy with genetically engineered HSV-1 expressing CCL2 and IL-12. Cancer Gene Ther. (2005) 12:359–68. doi: 10.1038/sj.cgt.7700784 15678154

[93] Tian L Xu B Chen Y Li Z Wang J Zhang J. Specific targeting of glioblastoma with an oncolytic virus expressing a cetuximab-CCL5 fusion protein via innate and adaptive immunity. Nat Cancer. (2022) 3:1318–35. doi: 10.1038/s43018-022-00448-0 PMC10150871 36357700

[94] Huang S Hu H Tang G Liu K Luo Z Zeng W. An oncolytic herpes simplex virus type 1 strain expressing a single-chain variable region antibody fragment against PD-1 and a PI3K inhibitor synergize to elicit antitumor immunity in ovarian cancer. Arch Virol. (2023) 168:128. doi: 10.1007/s00705-023-05754-1 37002434

[95] Ju F Luo Y Lin C Jia X Xu Z Tian R. Oncolytic virus expressing PD-1 inhibitors activates a collaborative intratumoral immune response to control tumor and synergizes with CTLA-4 or TIM-3 blockade. J Immunother Cancer. (2022) 10:e004762. doi: 10.1136/jitc-2022-004762 PMC9189843 35688558

[96] Aghi M Chou TC Suling K Breakefield XO Chiocca EA. Multimodal cancer treatment mediated by a replicating oncolytic virus that delivers the oxazaphosphorine/rat cytochrome P450 2B1 and ganciclovir/herpes simplex virus thymidine kinase gene therapies. Cancer Res. (1999) 59:3861–5. 10463570

[97] Shimizu K Kahramanian A Jabbar MADA Turna Demir F Gokyer D Uthamacumaran A. Photodynamic augmentation of oncolytic virus therapy for central nervous system Malignancies. Cancer Lett. (2023) 572:216363. doi: 10.1016/j.canlet.2023.216363 37619813 PMC10529118

[98] Broberg E Setala N Roytta M Salmi A Eralinna JP He B. Expression of interleukin-4 but not of interleukin-10 from a replicative herpes simplex virus type 1 viral vector precludes experimental allergic encephalomyelitis. Gene Ther. (2001) 8:769–77. doi: 10.1038/sj.gt.3301465 11420640

[99] Hotta Y Kasuya H Bustos I Naoe Y Ichinose T Tanaka M. Curative effect of HF10 on liver and peritoneal metastasis mediated by host antitumor immunity. Oncolytic Virother. (2017) 6:31–8. doi: 10.2147/OV.S127179 PMC5357077 28331843

[100] Purves FC Spector D Roizman B. The herpes simplex virus 1 protein kinase encoded by the US3 gene mediates posttranslational modification of the phosphoprotein encoded by the UL34 gene. J Virol. (1991) 65:5757–64. doi: 10.1128/JVI.65.11.5757-5764.1991 PMC250236 1656069

[101] Kanai R Wakimoto H Martuza RL Rabkin SD. A novel oncolytic herpes simplex virus that synergizes with phosphoinositide 3-kinase/Akt pathway inhibitors to target glioblastoma stem cells. Clin Cancer Res. (2011) 17:3686–96. doi: 10.1158/1078-0432.CCR-10-3142 PMC3107877 21505062

[102] Martuza RL Malick A Markert JM Ruffner KL Coen DM. Experimental therapy of human glioma by means of a genetically engineered virus mutant. Science. (1991) 252:854–6. doi: 10.1126/science.1851332 1851332

[103] Haines BB Denslow A Grzesik P Lee JS Farkaly T Hewett J. ONCR-177, an oncolytic HSV-1 designed to potently activate systemic antitumor immunity. Cancer Immunol Res. (2021) 9:291–308. doi: 10.1158/2326-6066.CIR-20-0609 33355229

[104] Passaro C Alayo Q De Laura I McNulty J Grauwet K Ito H. Arming an oncolytic herpes simplex virus type 1 with a single-chain fragment variable antibody against PD-1 for experimental glioblastoma therapy. Clin Cancer Res. (2019) 25:290–9. doi: 10.1158/1078-0432.CCR-18-2311 PMC6800097 30279232

[105] Chase M Chung RY Chiocca EA. An oncolytic viral mutant that delivers the CYP2B1 transgene and augments cyclophosphamide chemotherapy. Nat Biotechnol. (1998) 16:444–8. doi: 10.1038/nbt0598-444 9592392

[106] Currier MA Gillespie RA Sawtell NM Mahller YY Stroup G Collins MH. Efficacy and safety of the oncolytic herpes simplex virus rRp450 alone and combined with cyclophosphamide. Mol Ther. (2008) 16:879–85. doi: 10.1038/mt.2008.49 PMC2860295 18388918

[107] Studebaker AW Hutzen BJ Pierson CR Haworth KB Cripe TP Jackson EM. Oncolytic herpes virus rRp450 shows efficacy in orthotopic xenograft group 3/4 medulloblastomas and atypical teratoid/rhabdoid tumors. Mol Ther-Oncolytics. (2017) 6:22–30. doi: 10.1016/j.omto.2017.05.005 28649600 PMC5472147

[108] Pawlik TM Nakamura H Mullen JT Kasuya H Yoon SS Chandrasekhar S. Prodrug bioactivation and oncolysis of diffuse liver metastases by a herpes simplex virus 1 mutant that expresses the CYP2B1 transgene. Cancer-Am Cancer Soc. (2002) 95:1171–81. doi: 10.1002/cncr.10776 12209705

[109] Wang L Ning J Wakimoto H Wu S Wu C Humphrey MR. Oncolytic herpes simplex virus and PI3K inhibitor BKM120 synergize to promote killing of prostate cancer stem-like cells. Mol Ther-Oncolytics. (2019) 13:58–66. doi: 10.1016/j.omto.2019.03.008 31016228 PMC6468160

[110] Liu J Wang X Li Z Gao S Mao L Dai J. Neoadjuvant oncolytic virus orienx010 and toripalimab in resectable acral melanoma: a phase Ib trial. Signal Transduct Tar. (2024) 9:318. doi: 10.1038/s41392-024-02029-2 PMC11582582 39572525

[111] Barker CA D’Angelo SP Wasilewski G Steckler AM Lian M Zhang Z. A phase II randomized trial of talimogene laherparepvec oncolytic immunotherapy with or without radiotherapy for patients with cutaneous metastases from solid tumors. Radiother Oncol. (2024) 200:110478. doi: 10.1016/j.radonc.2024.110478 39159678 PMC11438562

[112] Robert C Gastman B Gogas H Rutkowski P Long GV Chaney MF. Open-label, phase II study of talimogene laherparepvec plus pembrolizumab for the treatment of advanced melanoma that progressed on prior anti-PD-1 therapy: MASTERKEY-115. Eur J Cancer. (2024) 207:114120. doi: 10.1016/j.ejca.2024.114120 38870745

[113] Ling AL Solomon IH Landivar AM Nakashima H Woods JK Santos A. Clinical trial links oncolytic immunoactivation to survival in glioblastoma. Nature. (2023) 623:157–66. doi: 10.1038/s41586-023-06623-2 PMC10620094 37853118

[114] Dummer R Gyorki DE Hyngstrom JR Ning M Lawrence T Ross MI. Final 5-year follow-up results evaluating neoadjuvant talimogene laherparepvec plus surgery in advanced melanoma: A randomized clinical trial. JAMA Oncol. (2023) 9:1457–9. doi: 10.1001/jamaoncol.2023.2789 PMC10416083 37561473

[115] Chesney JA Puzanov I Collichio FA Singh P Milhem MM Glaspy J. Talimogene laherparepvec in combination with ipilimumab versus ipilimumab alone for advanced melanoma: 5-year final analysis of a multicenter, randomized, open-label, phase II trial. J Immunother Cancer. (2023) 11:e006270. doi: 10.1136/jitc-2022-006270 PMC10163510 37142291

[116] Schwarze JK Tijtgat J Awada G Cras L Vasaturo A Bagnall C. Intratumoral administration of CD1c (BDCA-1)(+) and CD141 (BDCA-3)(+) myeloid dendritic cells in combination with talimogene laherparepvec in immune checkpoint blockade refractory advanced melanoma patients: a phase I clinical trial. J Immunother Cancer. (2022) 10:e005141. doi: 10.1136/jitc-2022-005141 PMC9486335 36113895

[117] Chesney JA Ribas A Long GV Kirkwood JM Dummer R Puzanov I. Randomized, double-blind, placebo-controlled, global phase III trial of talimogene laherparepvec combined with pembrolizumab for advanced melanoma. J Clin Oncol. (2023) 41:528–40. doi: 10.1200/JCO.22.00343 PMC9870217 35998300

[118] Todo T Ito H Ino Y Ohtsu H Ota Y Shibahara J. Intratumoral oncolytic herpes virus G47Δ for residual or recurrent glioblastoma: a phase 2 trial. Nat Med. (2022) 28:1630–9. doi: 10.1038/s41591-022-01897-x PMC9388376 35864254

[119] Todo T Ino Y Ohtsu H Shibahara J Tanaka M. A phase I/II study of triple-mutated oncolytic herpes virus G47Δ in patients with progressive glioblastoma. Nat Commun. (2022) 13:4119. doi: 10.1038/s41467-022-31262-y 35864115 PMC9304402

[120] Yamazaki N Isei T Kiyohara Y Koga H Kojima T Takenouchi T. A phase I study of the safety and efficacy of talimogene laherparepvec in Japanese patients with advanced melanoma. Cancer Sci. (2022) 113:2798–806. doi: 10.1111/cas.15450 PMC9357627 35656636

[121] Kai M Marx AN Liu DD Shen Y Gao H Reuben JM. A phase II study of talimogene laherparepvec for patients with inoperable locoregional recurrence of breast cancer. Sci Rep-Uk. (2021) 11:22242. doi: 10.1038/s41598-021-01473-2 PMC8593093 34782633

[122] Dummer R Gyorki DE Hyngstrom J Berger AC Conry R Demidov L. Neoadjuvant talimogene laherparepvec plus surgery versus surgery alone for resectable stage IIIB-IVM1a melanoma: a randomized, open-label, phase 2 trial. Nat Med. (2021) 27:1789–96. doi: 10.1038/s41591-021-01510-7 34608333

[123] Monga V Miller BJ Tanas M Boukhar S Allen B Anderson C. Intratumoral talimogene laherparepvec injection with concurrent preoperative radiation in patients with locally advanced soft-tissue sarcoma of the trunk and extremities: phase IB/II trial. J Immunother Cancer. (2021) 9:e003119. doi: 10.1136/jitc-2021-003119 PMC8327848 34330766

[124] Malvehy J Samoylenko I SChadendorf D Gutzmer R Grob J Sacco JJ. Talimogene laherparepvec upregulates immune-cell populations in non-injected lesions: findings from a phase II, multicenter, open-label study in patients with stage IIIB-IVM1c melanoma. J Immunother Cancer. (2021) 9:e001621. doi: 10.1136/jitc-2020-001621 PMC8011715 33785610

[125] Soliman H Hogue D Han H Mooney B Costa R Lee MC. A phase I trial of talimogene laherparepvec in combination with neoadjuvant chemotherapy for the treatment of nonmetastatic triple-negative breast cancer. Clin Cancer Res. (2021) 27:1012–8. doi: 10.1158/1078-0432.CCR-20-3105 33219014

[126] Harrington KJ Kong A Mach N Chesney JA Fernandez BC Rischin D. Talimogene laherparepvec and pembrolizumab in recurrent or metastatic squamous cell carcinoma of the head and neck (MASTERKEY-232): A multicenter, phase 1b study. Clin Cancer Res. (2020) 26:5153–61. doi: 10.1158/1078-0432.CCR-20-1170 32669371

[127] Kelly CM Antonescu CR Bowler T Munhoz R Chi P Dickson MA. Objective response rate among patients with locally advanced or metastatic sarcoma treated with talimogene laherparepvec in combination with pembrolizumab: A phase 2 clinical trial. JAMA Oncol. (2020) 6:402–8. doi: 10.1001/jamaoncol.2019.6152 PMC6990941 31971541

[128] Streby KA Currier MA Triplet M Ott K Dishman DJ Vaughan MR. First-in-human intravenous seprehvir in young cancer patients: A phase 1 clinical trial. Mol Ther. (2019) 27:1930–8. doi: 10.1016/j.ymthe.2019.08.020 PMC6838937 31570234

[129] Andtbacka RHI Amatruda T Nemunaitis J Zager JS Walker J Chesney JA. Biodistribution, shedding, and transmissibility of the oncolytic virus talimogene laherparepvec in patients with melanoma. Ebiomedicine. (2019) 47:89–97. doi: 10.1016/j.ebiom.2019.07.066 31409575 PMC6796514

[130] Andtbacka RHI Collichio F Harrington KJ Middleton MR Downey G Öhrling K. Final analyses of OPTiM: a randomized phase III trial of talimogene laherparepvec versus granulocyte-macrophage colony-stimulating factor in unresectable stage III-IV melanoma. J Immunother Cancer. (2019) 7:145. doi: 10.1186/s40425-019-0623-z 31171039 PMC6554874

[131] Hirooka Y Kasuya H Ishikawa T Kawashima H Ohno E Villalobos IB. A Phase I clinical trial of EUS-guided intratumoral injection of the oncolytic virus, HF10 for unresectable locally advanced pancreatic cancer. BMC Cancer. (2018) 18:596. doi: 10.1186/s12885-018-4453-z 29801474 PMC5970460

[132] Chesney J Puzanov I Collichio F Singh P Milhem MM Glaspy J. Randomized, open-label phase II study evaluating the efficacy and safety of talimogene laherparepvec in combination with ipilimumab versus ipilimumab alone in patients with advanced, unresectable melanoma. J Clin Oncol. (2018) 36:1658–67. doi: 10.1200/JCO.2017.73.7379 PMC6075852 28981385

[133] Waters AM Johnston JM Reddy AT Fiveash J Madan-Swain A Kachurak K. Rationale and design of a phase 1 clinical trial to evaluate HSV G207 alone or with a single radiation dose in children with progressive or recurrent Malignant supratentorial brain tumors. Hum Gene Ther Clin Dev. (2017) 28:7–16. doi: 10.1089/humc.2017.002 28319448 PMC5369388

[134] Andtbacka RHI Ross M Puzanov I Milhem M Collichio F Delman KA. Patterns of clinical response with talimogene laherparepvec (T-VEC) in patients with melanoma treated in the OPTiM phase III clinical trial. Ann Surg Oncol. (2016) 23:4169–77. doi: 10.1245/s10434-016-5286-0 PMC5090012 27342831

[135] Patel DM Foreman PM Nabors LB Riley KO Gillespie GY Markert JM. Design of a phase I clinical trial to evaluate M032, a genetically engineered HSV-1 expressing IL-12, in patients with recurrent/progressive glioblastoma multiforme, anaplastic astrocytoma, or gliosarcoma. Hum Gene Ther Clin Dev. (2016) 27:69–78. doi: 10.1089/humc.2016.031 27314913 PMC4932657

[136] Andtbacka RHI Kaufman HL Collichio F Amatruda T Senzer N Chesney J. Talimogene laherparepvec improves durable response rate in patients with advanced melanoma. J Clin Oncol. (2015) 33:2780–8. doi: 10.1200/JCO.2014.58.3377 26014293

[137] Kasuya H Kodera Y Nakao A Yamamura K Gewen T Zhiwen W. Phase I dose-escalation clinical trial of HF10 oncolytic herpes virus in 17 Japanese patients with advanced cancer. Hepatogastroenterology. (2014) 61:599–605. 26176043

[138] Markert JM Razdan SN Kuo H Cantor A Knoll A Karrasch M. A phase 1 trial of oncolytic HSV-1, G207, given in combination with radiation for recurrent GBM demonstrates safety and radiographic responses. Mol Ther. (2014) 22:1048–55. doi: 10.1038/mt.2014.22 PMC4015243 24572293

[139] Harrington KJ Hingorani M Tanay MA Hickey J Bhide SA Clarke PM. Phase I/II study of oncolytic HSV GM-CSF in combination with radiotherapy and cisplatin in untreated stage III/IV squamous cell cancer of the head and neck. Clin Cancer Res. (2010) 16:4005–15. doi: 10.1158/1078-0432.CCR-10-0196 20670951

[140] Kaufman HL Bines SD. OPTIM trial: a Phase III trial of an oncolytic herpes virus encoding GM-CSF for unresectable stage III or IV melanoma. Future Oncol. (2010) 6:941–9. doi: 10.2217/fon.10.66 20528232

[141] Senzer NN Kaufman HL Amatruda T Nemunaitis M Reid T Daniels G. Phase II clinical trial of a granulocyte-macrophage colony-stimulating factor-encoding, second-generation oncolytic herpesvirus in patients with unresectable metastatic melanoma. J Clin Oncol. (2009) 27:5763–71. doi: 10.1200/JCO.2009.24.3675 19884534

[142] Kemeny N Brown K Covey A Kim T Bhargava A Brody L. open-label, dose-escalating study of a genetically engineered herpes simplex virus, NV1020, in subjects with metastatic colorectal carcinoma to the liver. Hum Gene Ther. (2006) 17:1214–24. doi: 10.1089/hum.2006.17.1214 17107303

[143] Filley AC Dey M. Immune system, friend or foe of oncolytic virotherapy? Front Oncol. (2017) 7:106. doi: 10.3389/fonc.2017.00106 28589085 PMC5440545

[144] Zheng N Fang J Xue G Wang Z Li X Zhou M. Induction of tumor cell autosis by myxoma virus-infected CAR-T and TCR-T cells to overcome primary and acquired resistance. Cancer Cell. (2022) 40:973–85. doi: 10.1016/j.ccell.2022.08.001 PMC9489043 36027915

[145] Evgin L Kottke T Tonne J Thompson J Huff AL van Vloten J. Oncolytic virus-mediated expansion of dual-specific CAR T cells improves efficacy against solid tumors in mice. Sci Transl Med. (2022) 14:eabn2231. doi: 10.1126/scitranslmed.abn2231 35417192 PMC9297825

[146] Guan J Sun K Guerrero CA Zheng J Xu Y Mathur S. A phase 2 study of *in situ* oncolytic virus therapy and stereotactic body radiation therapy followed by pembrolizumab in metastatic non-small cell lung cancer. Int J Radiat Oncol. (2024) 118:1531–40. doi: 10.1016/j.ijrobp.2023.08.044 37625523

[147] Chen NG Yu YA Zhang Q Szalay AA. Replication efficiency of oncolytic vaccinia virus in cell cultures prognosticates the virulence and antitumor efficacy in mice. J Transl Med. (2011) 9:164. doi: 10.1186/1479-5876-9-164 21951588 PMC3192684

